# Comparative Analysis of the Composition and Active Property Evaluation of Certain Essential Oils to Assess their Potential Applications in Active Food Packaging

**DOI:** 10.3390/ma10010045

**Published:** 2017-01-07

**Authors:** Cornelia Vasile, Morten Sivertsvik, Amalia Carmen Miteluţ, Mihai Adrian Brebu, Elena Stoleru, Jan Thomas Rosnes, Elisabeta Elena Tănase, Waqas Khan, Daniela Pamfil, Călina Petruţa Cornea, Anamaria Irimia, Mona Elena Popa

**Affiliations:** 1 Physical Chemistry of Polymers Department, “Petru Poni” Institute of Macromolecular Chemistry Romanian Academy, 41A, Gr. Ghica Voda Alley, Iasi 700487, Romania; bmihai@icmpp.ro (M.A.B.); elena.paslaru@icmpp.ro (E.S.); pamfil.daniela@icmpp.ro (D.P.); anamaria.sdrobis@icmpp.ro (A.I.); 2 Nofima AS, Department of Processing Technology, Muninbakken 9-13, Tromsø 9291, Norway; thomas.rosnes@nofima.no; 3 Faculty of Biotechnology, University of Agronomic Sciences and Veterinary Medicine of Bucharest, 59 Mărăşti Blvd, District 1, Bucharest 011464, Romania; amaliamitelut@yahoo.com (A.C.M.); elena.eli.tanase@gmail.com (E.E.T.); pccornea@yahoo.com (C.P.C.); pandry2002@yahoo.com (M.E.P.); 4 Department of Biological Chemistry, University of Stavanger, Stavanger 4036, Norway; khanwaqas2006@gmail.com

**Keywords:** essential oils, antifungal, antimicrobial, antioxidant, spoilage fungi

## Abstract

The antifungal, antibacterial, and antioxidant activity of four commercial essential oils (EOs) (thyme, clove, rosemary, and tea tree) from Romanian production were studied in order to assess them as bioactive compounds for active food packaging applications. The chemical composition of the oils was determined with the Folin–Ciocâlteu method and gas chromatography coupled with mass spectrometry and flame ionization detectors, and it was found that they respect the AFNOR/ISO standard limits. The EOs were tested against three food spoilage fungi—*Fusarium graminearum*, *Penicillium corylophilum,* and *Aspergillus brasiliensis*—and three potential pathogenic food bacteria—*Staphylococcus aureus*, *Escherichia coli,* and *Listeria monocytogenes*—using the disc diffusion method. It was found that the EOs of thyme, clove, and tea tree can be used as antimicrobial agents against the tested fungi and bacteria, thyme having the highest inhibitory effect. Concerning antioxidant activity determined by 2,2-diphenyl-1-picrylhydrazyl (DPPH) and 2,2’-azino-bis 3-ethylbenzthiazoline-6-sulfonic acid (ABTS) methods, it has been established that the clove oil exhibits the highest activity because of its high phenolic content. Promising results were obtained by their incorporation into chitosan emulsions and films, which show potential for food packaging. Therefore, these essential oils could be suitable alternatives to chemical additives, satisfying the consumer demand for naturally preserved food products ensuring its safety.

## 1. Introduction

Consumers demand high quality foods with minimal changes in nutritional properties. A minimal amount of synthetic additives combined with a suitable packaging technology that retains or creates desirable food qualities or reduces undesirable changes in food due to microbial activity is therefore a goal of food manufacturers [1]. New processes must be designed to meet the required food product safety or shelf-life demands, and additional hurdles for microorganisms should be introduced. World Health Organization (WHO) reports [2] in recent years estimate that 30% of people in industrialized countries suffer from a food-borne disease each year. Reducing or eliminating food-borne pathogens via “green” consumerism concomitantly with low salt consumption to diminish the incidence of cardiovascular diseases is increasingly becoming a public health concern. On the other hand, antimicrobial resistance affects all areas of health, as many medicinal procedures are related to antibiotics [3].

Therefore, new methods and additives should be found to prolong service life and to improve the safety of foods. Recently, effective preventive measures and intelligent preservation methods have been put into place to reduce food spoilage, increase safety, and prolong food shelf-life. One of these methods is bioactive packaging by the use of natural compounds with multifunctional properties both to achieve the protection of food and to improve the health of consumers. The concern regarding safety issues of the synthetic antimicrobial agents has led to the use of essential oils (EOs), which represent eco-friendly alternatives to chemicals. Essential oils (also called volatile oils) are oily liquids obtained from plant materials (flowers, buds, seeds, leaves, twigs, bark, herbs, wood, fruits, and roots). Plant-derived essential oils are complex mixtures of natural volatile compounds resulting from the plant secondary metabolism and extracted from vegetable materials by expression (i.e., “cold pressing”), fermentation, enfleurage, or extraction, but the method of distillation with water or steam is the most commonly used for the commercial production of EOs. Essential oils contain important classes of compounds such as monoterpenes (C_10_ hydrocarbons based on 2 isoprene units), phenylpropanoides (C_6_ aromatic compounds with C_3_ side chains), sesquiterpenes (C_15_ hydrocarbons based on 3 isoprene units), diterpenes (C_20_), triterpenes (C_30_) and their oxygenated derivatives, and phenolic compounds (such as thymol and carvacrol). Due to their versatile content, essential oils constitute a rich source of biologically active compounds possessing antimicrobial, antibacterial, antifungal, antioxidant, antiviral, antimycotic, antitoxigenic, antiparasitic, antibiotic, and antiseptic properties and insecticidal activities; therefore, they are useful in a wide range of applications [4,5].

Each of the above-mentioned constituents contributes to beneficial or adverse effects; therefore, it is very important to know as much as possible about the composition and properties of EOs, with each study bringing new highlights on the advantages and disadvantages they offer.

There are many studies on the characterization of volatile compound composition and the antimicrobial and antioxidant activities of various selected groups of essential oils. Bozin et al. characterized Lamiaceae species and the antimicrobial and antioxidant activities of the oils of *Ocimum basilicum* L., *Origanum vulgare* L., and *Thymus vulgaris* L. [6], and the chemical constituents of four populations of *Piper aduncum* L. from Distrito Federal, Brazil [7], were identified. Many other essential oils have been characterized, namely, sweet lime (*Citrus limetta* Risso) [8], *Chenopodium ambrosioides*, *Philodendron bipinnatifidum* [9], cinnamon oil, eucalyptus oil, lemongrass oil, peppermint oil, citronella oil, turpentine oil [10], citronella oil [11], *Tithonia diversifolia* (Hemsl.) A. Gray [12], *O. basilicum* L. from Italy [13], and Iranian geranium oil [14]. The composition of two species of mint (*Mentha suaveolens* Ehrh. and *Mentha rotundifolia*) grown in Orăştie-Romania has been comparatively examinated [15]. From the 21 plant essential oils (cinnamon, clove, geranium, lemon, lime, orange and rosemary, aniseed, eucalyptus, and camphor) tested against six bacterial species four Gram-negative bacteria (*Escherichia coli*, *Klebsiella pneumoniae*, *Pseudomonas aeruginosa*, and *Proteus vulgaris*) and two Gram-positive bacteria *Bacillus subtilis* and *Staphylococcus aureus* [16], 19 oils showed antibacterial activity.

Consumers are worried about the presence of chemical preservatives, which can lead to benzoic acid by the decarboxylating action of some spoilage microorganisms, and this is considered the cause of many carcinogenic and teratogenic attributes and residual toxicity. Therefore, the studies to find natural and socially acceptable preservatives receive increasing attention by screening the composition and the biological, antimicrobial, and antioxidant activities of plant extracts [17]. Essential oils can prevent fungal growth in food products, which may cause spoilage and result in a reduction in the quality and quantity of food (shelf-life). Most EOs applied directly onto food or in the vapor phase can reduce or stop the colony forming ability of molds. They are also regarded as safe (GRAS) and are accepted by the FDA and by consumers. By their potential antimicrobial/antifungal/antioxidant effects, EOs could be the answer to the current search for environmental solutions and to assuring the microbial safety of food products in active packaging applications [18,19].

In this study, four essential oils, namely, thyme (*Thymus vulgaris* L.), clove (*Eugenia caryophyllus* from dried floral buds of *Syzygium aromaticum*), rosemary (*Rosmarinus officinalis* L.), and tea tree (*Melaleuca alternifolia aetheroleum*) obtained from Romanian production (Fares Co., Orăştie, Romania) are intent to be used as components in bioactive food packaging. Therefore, as a first step, it should be worthwhile to comparatively evaluate the composition and antimicrobial/antioxidant activity of these four EOs. Antifungal and antibacterial activity against three target food spoilage fungi (*Aspergillus brasiliensis*, *Fusarium graminearum*, and *Penicillium corylophilum*) and three potential pathogenic food bacteria (*S. aureus*, *E. coli,* and *L. monocytogenes*) have been evaluated. The minimum inhibitory concentration (MIC) concentration was established in each case. The antioxidant activity was determined, and the most efficient oil for each type of activity was established. Preliminary tests on the EO encapsulation into chitosan films and their antimicrobial activity against the spoilage of beef meat showed promising results, a detailed presentation for which will be provided in a future paper.

## 2. Results and Discussion

### 2.1. Chemical Composition

#### 2.1.1. Phenolic Content

The phenolic content of extracts of many plants contributes significantly to their total antioxidant activity. The antioxidant feature of the investigated essential oils was determined by the phenolic compouds presence in their composition by the Folin–Ciocâlteu method [20].

The total concentration of phenolic compounds found in essential oil samples are presented in Table 1, and a decrease in phenolic content concentration in the following order was found: clove > thyme > tea tree > rosemary. Excepting the rosemary oil, all oils were found to have different phenolic levels, ranging from 0.034 to 1.136 mg·GAE/g·DW, which can play a vital role in the increase of food shelf-life. The clove oil has the highest content of phenolic compounds.

#### 2.1.2. GC-MSD and GC-FID Analysis

The composition (including both main components and those in low amount but with significant biological/therapeutic effects) of the essential oils varies depending on the geographical position, the plant’s origin and species, harvest time, distillation/extraction procedure, etc. [17]. Additionally, the composition of the essential oils and consequently their biological/therapeutic activities depend on the combination and ratio of their numerous different components. Gas chromatography coupled with mass spectrometry (GC-MSD) and flame ionization (GC-FID) detectors was used to determine the quality and quantity of chemical compounds in the essential oils.

**Figure 1:**
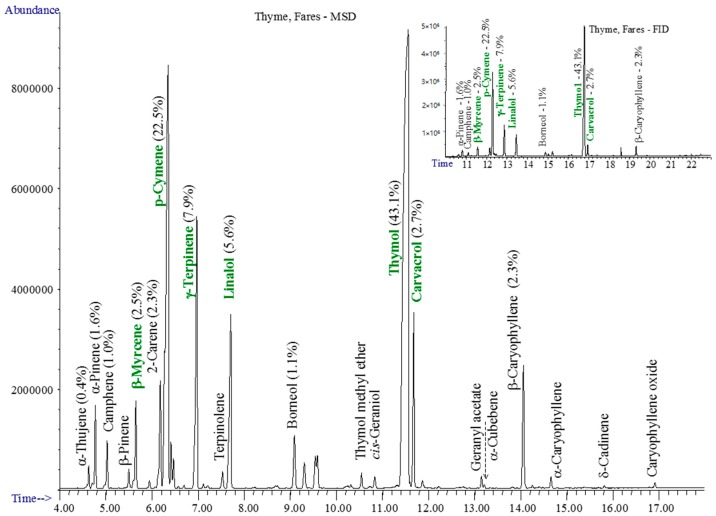
The GC-MSD and GC-FID (insert) chromatograms of thyme oil.

**Figure 2:**
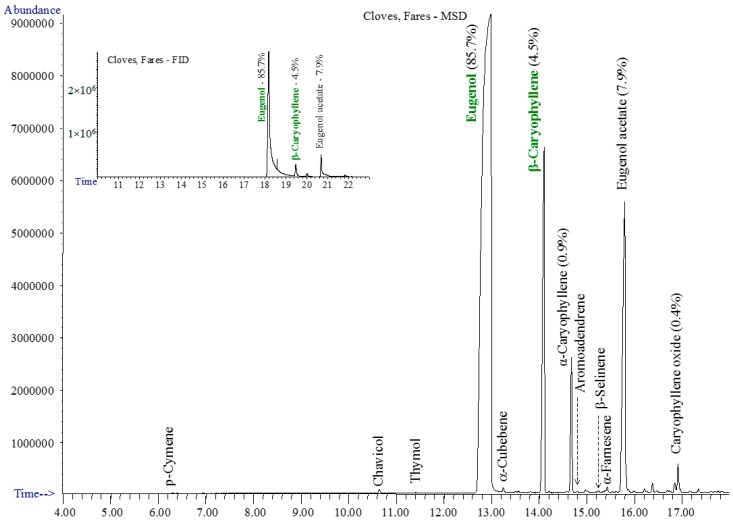
The GC-MSD and GC-FID (insert) chromatograms of clove oil.

**Figure 3:**
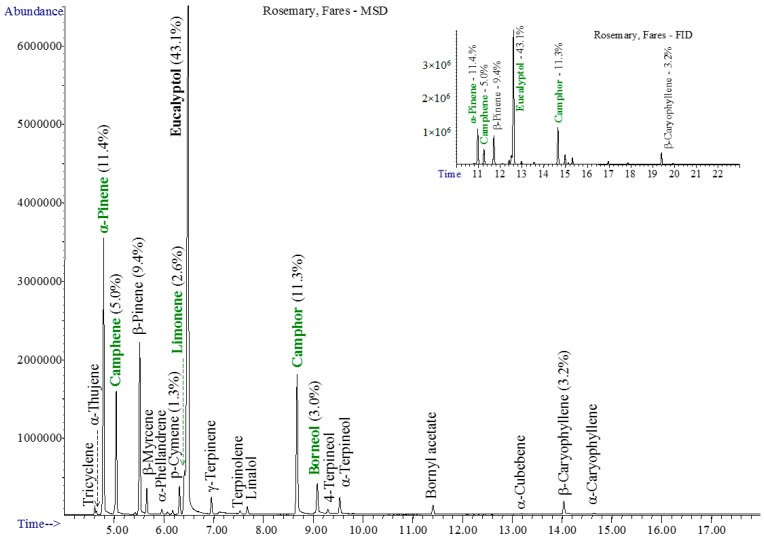
The GC-MSD and GC-FID (insert) chromatograms of rosemary oil.

**Figure 4:**
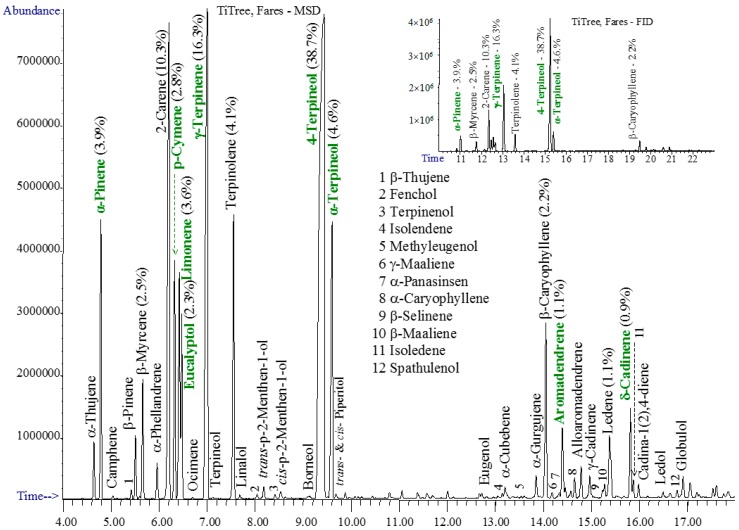
The GC-MSD and GC-FID (insert) chromatograms of tea tree oil.

The chromatograms of studied essential oils are shown in Figure 1, Figure 2, Figure 3 and Figure 4.

The essential thyme oil contains especially thymol, p-cymene, γ-terpinene, linalol, isothymol (carvacrol/biosol), and β-myrcene—Figure 1.

The clove essential oil had the simplest composition, based on eugenol/eugenol acetate and β-/α-caryophyllene, accompanied by several other sesquiterpenes—Figure 2.

The rosemary oil is rich in light monoterpenes, containing mainly eucalyptol, camphor, α-/β-pinene, camphene, borneol, and limonene—Figure 3.

The tea tree essential oil contained both light monoterpenes and numerous sesquiterpenes. The main compound is 4-terpineol, followed by γ-terpinene, 2-carene, α-terpineol, α-terpinene, α-pinene, o-cymene, limonene, β-caryophyllene, eucalyptol, and β-myrcene—Figure 4. Standards ask for α-terpinene (5%–13%), which was not found in our tee tree sample, instead 2-carene was found in high amounts of about 10%. Aromadendrene and δ-cadinene, mentioned by standards in amounts varying from traces up to 7%–8% were confirmed in the studied sample.

The main classes of compounds identified in the studied essential oils are presented in Table 2. Under the mentioned analysis parameters, only volatile compounds up to the level of sesquiterpenes were detected.

Table 3 shows the quantitative composition (based on Gas Chromatographz with Flame Ioniyation Detector (GC-FID) of the analyzed essential oils. The GC-FID analysis was performed on several different samples obtained from the same company, i.e., Fares, Orăştie-Romania, and no relevant difference was found between them. The results are reproducible.

The composition of the four essential oils is within the limits of AFNOR (Association French Normalization Organization Regulation)/ISO standards [21,22,23,24,25], except for a few differences, which could be considered within the error limits of the analysis. Deviation in the composition from standard values may not interfere with the therapeutic properties of the essential oils; however, if oils respect the standard limits, it is safer to consider them usable according to the general practice.

The ORAC index shows the antioxidant capacity of the oils. According to databases, the antioxidant capacity is highest for clove essential oil, followed at a high distance by thyme oil, while the antioxidant capacity for tea tree is smaller than those of other essential oils. These statements are in accordance with results obtained by the Folin–Ciocâlteu method.

Based on the gas chromatography analysis coupled with mass spectrometry and flame ionisation detectors, the commercial essential oils of thyme (*Thymus vulgaris* L.), clove (*Eugenia caryophyllus*), rosemary (*Rosmarinus officinalis* L.), and tea tree (*Melaleuca alternifolia aetheroleum*) were found to have compositions within the limits of the AFNOR/ISO standards.

Most of the obtained results related to the composition of the studied essential oils are in accordance with those found by other authors and constitute support for the explanation of their potential biological activity spectrum [28,29,30,31,32,33,34,35,36,37,38,39,40,41,42,43,44]. Thyme essential oil (TEO) is obtained from *Thymus vulgaris* L. and exhibits antimicrobial effects due to its constituents. Omidbeygi et al. [28] found that the major compounds of TEO are thymol, carvacrol with similar chemical structures, linalool, and ρ-cymene [28,29]. The presence of thymol (2-Isopropyl-5-methylphenol) and carvacrol enhanced the TEO antimicrobial activity [30,31,32]. Clove essential oil (*Caryophylli aetheroleum*) (CEO) isolated from the dry floral buds of *Syzygium aromaticum*, belonging to the *Myrtaceae* family has been used for its antimicrobial activity. Goni [33], Shao [34], and Sebaaly [35] established that the CEO is composed mainly of phenylpropanoides such as eugenol, β-caryophyllene, and the eugenyl acetate [33,34,35,36].

Jiang et al. [37] found that the rosemary essential oil (*Rosmarinus officinalis* L.) (REO) can be used in the food industry as a flavoring agent and preservative because of its antimicrobial and antioxidant properties [37]. Among the terpenes found in the composition of REO, the main components are the following: cineole, camphor, α-pinene, camphene, and α-terpineol [38,39].

Sánchez-González et al. [33,40] established that the essential oil of *Melaleuca alternifolia,* also known as tea tree essential oil (TTO) is composed of terpene, mainly monoterpenes, sesquiterpenes, and tertiary alcohols [33,40,41,42,43]. The main components of TTO are terpineol, cineol, pinen, and terpinen and demonstrated antimicrobial activity [40,44].

### 2.2. Antifungal Activity of the Tested Essential Oils

**Figure 5:**
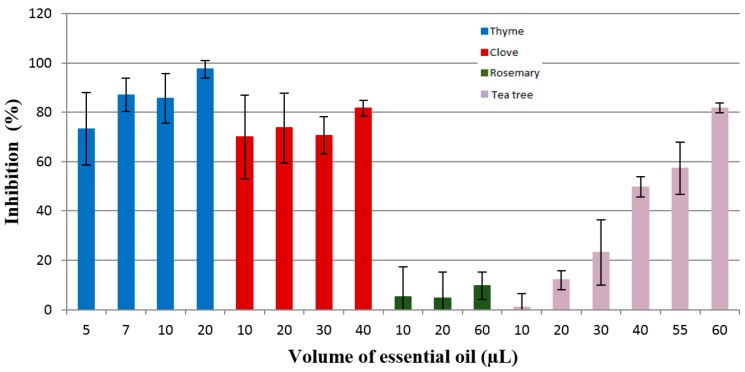
Inhibition of the *Fusarium graminearum* G87 growth by the thyme, clove, rosemary, and tea tree essential oils.

**Figure 6:**
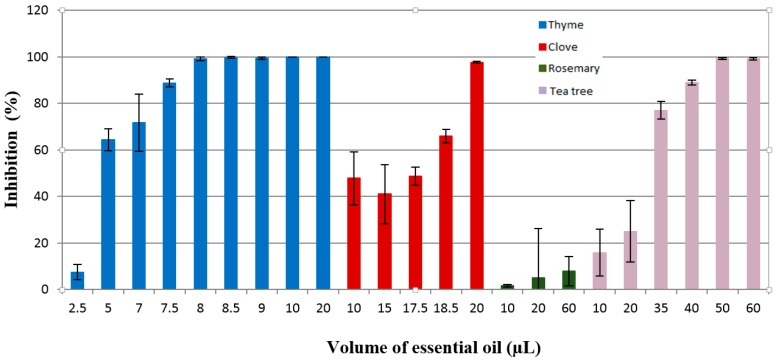
Inhibition of the *Penicillium corylophilum* CBMF1 growth by the thyme, clove, rosemary, and tea tree essential oils.

**Figure 7:**
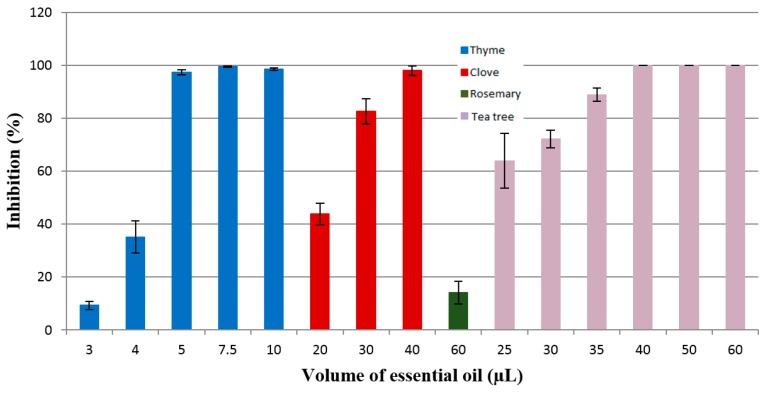
Inhibition of the *Aspergillus brasiliensis* ATCC 16404 growth by the thyme, clove, rosemary, and tea tree essential oils.

The antifungal activity of the essential oils against the three fungi strains examined was assessed by the percentage of the inhibition rate (*IR*%) and minimum inhibitory concentration (MIC). In order to evaluate the quantity of essential oils needed to be used, a preliminary investigation was carried out with a volume of 10 µL and 20 µL of each of them. After this screening, the maximum concentration used was 60 µL, while the minimum volume used was 2.5 µL. The results presented in Figure 5, Figure 6 and Figure 7 for the antifungal activity of the essential oils tested are the ones from the 7th day of analyses. From all tested oils, the most effective were the essential oils of clove, thyme, and tea tree. The efficacy of these essential oils can be attributed to the terpenes and phenylpropanoides present in their composition. The research studies demonstrated that phenolic compounds are responsible for the antifungal potential of these oils.

#### 2.2.1. *Fusarium Graminearum* G87

*Fusarium graminearum*, being a plant pathogen, is a fungus commonly found in cereal grains (mostly wheat and barley). This pathogen has the potential to produce mycotoxines (deoxynivalenol and zearalonene) that have a negative impact on human and animal health [45]. The contamination of cereals by this toxigenic mold produces major economic impacts in agriculture.

The essential oils selected were tested against *Fusarium graminearum*, and the results are presented in Figure 5.

The fungal growth inhibition induced by essential oils, as determined by the disc diffusion assay, was dependent mostly on the volume (varying from 5 µL to 60 µL) and nature of the essential oils. According to the results presented in Figure 5, it can be noticed that the essential oils tested had antifungal activity against *Fusarium graminearum* G87, but their efficacy is different. Thyme essential oil was the most effective, with an inhibition rate of over 80% at the lowest volume of essential oil used for the growth inhibition of this fungus, namely, 87.04% for 7 µL. Clove oil was also effective against the tested fungus, with an inhibition rate of 81.64% when using 40 µL. It is assumed that the high antifungal potential of clove essential oil is due to its active principle, eugenol (85.7%). The results are in accordance with the study of Abbaszadeh et al. [46] regarding the effectiveness of eugenol against pathogenic fungi, which demonstrated that this phenylpropanoide had antifungal activity against all tested fungi. The antifungal activity was in direct ratio with the concentration of eugenol added to the media. In a study made by Marin et al. [47] who tested the efficacy of cinnamon, clove, oregano, palmarosa, and lemongrass oils against the mycotoxines produced by *Fusarium graminearum*, it was shown that clove essential oils was the most efficient against zearalenone and deoxynivalenol release.

Rosemary essential oil exhibits antifungal activity, but this was very low. For a volume of 60 µL of this oil, the inhibition was below 10%. Tea tree essential oil had an inhibition rate higher than 80% against *F. graminearum* G87 only for the highest volume used in this study (60 µL).

#### 2.2.2. *Penicillium Corylophilum* CBMF1

*Penicillium corylophilum* is a fungus that may cause the spoilage of bakery products; for this judgment, the essential oils selected were used to determine their antifungal activity against this mold.

Figure 6 shows the inhibition manifested by the tested essential oils against *Penicillium corylophilum* growth.

The effectiveness of thyme oil as an antifungal agent can be noticed from the data presented in Figure 6. As can be seen, thyme essential oil is more active than clove essential oil, showing an inhibition rate of 100% at 10 µL, while clove oil was less effective (*IR* = 47.6%) at a volume of 10 µL. As well, tea tree essential oil has antifungal properties, but when a higher oil volume is used. It produced an inhibition of 89.04% for a quantity of 40 µL. It is considered that the antifungal activity of tea tree oil was significantly affected by the presence of high content of γ-terpinene (16.3%), 4-terpineol (38.7%), and α-terpineol (4.6%). Previous studies showed that tea tree oil has antifungal activity against *Botrytis cinerea* and *Rhizopus stolonifer* under in vitro conditions, inhibiting the spore germination and mycelial growth [43].

For the highest volume used in this study (60 µL), rosemary oil did not inhibit the fungal growth of *Penicillium corylophilum* CBMF1, the IR being below 10%.

#### 2.2.3. *Aspergillus Brasiliensis* ATCC 16404

*Aspergillus brasiliensis* is a black mold that can spoil food products, especially fruits and vegetables [48].

The inhibition rate of the tested essential oils (thyme oil, clove oil, rosemary oil, tea tree oil) against *Aspergillus brasiliensis* ATCC 16404 is presented in Figure 7. The results presented evidenced that thyme oil was effective against *A. brasiliensis* ATCC 16404 at a volume of 5 µL (*IR* = 97.43%), while clove oil shows antifungal activity at a volume of 30 µL (*IR* = 82.64%). The strong antifungal activity of thyme oil is attributed to phenolic compounds carvacrol (2.75%) and thymol (43.1%), while the antimicrobial effectiveness of clove oil is associated with the activity of eugenol (85.7%), β-caryophyllene (4.5%), and eugenol acetate (7.9%). Similar studies were conducted by Abbaszadeh et al. [46], who tested the antifungal efficiency of thymol, carvacrol, eugenol, and menthol on growth inhibition of some important food-borne pathogens. The results showed that thymol and carvacrol inhibited the fungal growth of *Cladosporium* spp., *Aspergillus* spp., *Fusarium oxysporum*, *Botrytis cinerea*, *Penicillium* spp., *Alternaria alternata*, and *Rhizopus oryzae*, and the inhibition growth was dependent on the concentration used.

The essential oil of rosemary showed an inhibition rate of 14.22% for the volume of 60 µL. Jiang et al. [37] tested the antimicrobial activity of rosemary essential oil and its main components, α-pinene and 1.8-cineole, against Gram-positive and Gram-negative bacteria and fungi (*Candida albicans*, *Aspergillus niger*). This study showed that the antimicrobial activity of rosemary essential oil was superior to its active compounds, and the essential oil is more active against all the bacteria used in this research compared to the tested fungi, concluding that the synergism between its compounds determine the antifungal activity.

Tea tree essential oil inhibited the growth of the tested fungus with an inhibition rate of 88.90% for a volume of 35 µL.

Comparing antifungal activity results of the studied essential oils, the following decreasing order of activity is evident: thyme oil > clove oil > tea tree oil >> rosemary oil. Concerning the sensitivity to various fungi, the thyme oil and tea tree oil are very effective in the inhibition of the *Aspergillus brasiliensis,* as is clove oil in the inhibition of the *Penicillium corylophilum.* The rosemary oil is less effective as an antifungal agent but shows some activity against *Aspergillus brasiliensis.* However, other studies have evidenced that high-quality rosemary oil has antitumor, antifungal, and antiparasitic effects [49]. It has also analgesic, anticancer, anticatarrhal, anti-infection, anti-inflammatory, and expectorant properties and stimulates the circulatory system [50].

### 2.3. Minimum Inhibitory Concentration

The minimum inhibitory concentrations of the tested essential oils (EOs of thyme, clove, rosemary, and tea tree oils) are presented in Table 4.

The data presented in Table 4 shows that, from all the essential oils tested in this study, rosemary oil did not show desirable results against the selected fungi.

The results evidenced that the most effective essential oil from the tested oils was thyme essential oil, which displayed strong antifungal activity with MIC values between 116.27 and 174.41 ppm. MIC values of clove, rosemary, and tea tree essential oils were found within the range of 465.11–1395.34 ppm.

### 2.4. Antibacterial Inhibition

**Figure 8:**
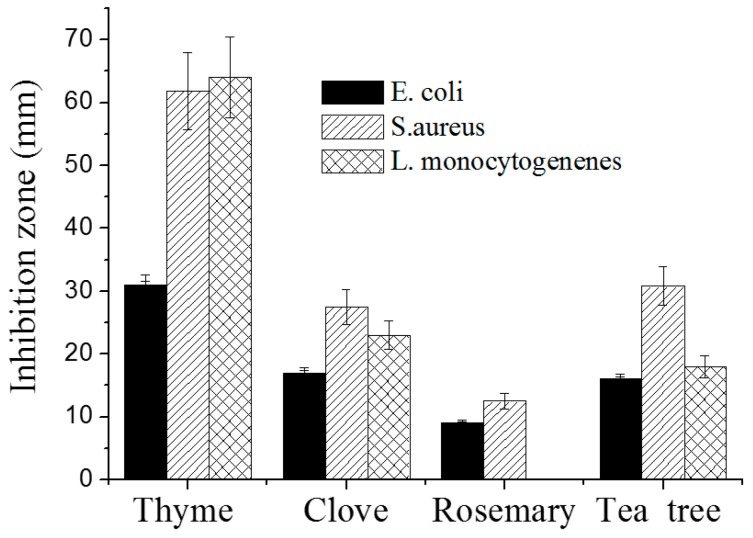
Antimicrobial activity of the four essential oils (EOs) (10 µL on each disc) against *S. aureus*, *E. coli,* and *L. monocytogenes* using the agar disc diffusion method on an Mueller–Hinten (MH) Plate.

The antibacterial effect of EOs against the growth of *S. aureus*, *E. coli*, and *L. monocytogenes* was established by the agar disc diffusion method, and the results are presented in Figure 8. On Mueller–Hinten (MH) agar plates, the inhibition zones of *S. aureus* are 64.7 ± 1.2 mm for thyme oil, 27.8 ± 3.4 mm for tea tree oil, 27.8 ± 4.0 mm for clove oil, and 12.8 ± 4.3 mm for rosemary oil. For *E. coli,* the inhibition zones are 35.5 ± 4.6 mm for thyme oil, 27.0 ± 3.4 mm for tea tree oil, 19.5 ± 0.5 mm for clove oil, and 15.1 ± 0.5 mm for rosemary oil. The diameters of the inhibition zones in the case of *L. monocytogenes* growth are of 69.5 ± 6.4 mm for thyme oil, 22.0 ± 2.8 mm for tea tree oil, and 28.5 ± 2.1 mm for clove oil. There is no inhibition zone for rosemary.

Thyme oil had the highest inhibition zones for the three test strains, followed by tea tree oil, and then by clove oil. Rosemary oil was the least active.

Thyme essential oil is strongly antimicrobial, antifungal, antiviral, antiparasitic, mainly due to the high content of thymol [49]. This is in accordance with other studies that reported that thyme essential oil is efficient against Gram-positive and Gram-negative bacteria [29,30] and fungi [28,51,52].

Due to its antimicrobial, antifungal, antivermin, and antiviral activity [34,35], clove essential oil is used as a preservative but also exhibits other effects with positive impacts on consumer health, including antiseptic, antihelmintic, anti-inflammatory, antispastic, carminative, anti-neuralgic, antiulcer, anti-thrombotic, anticancerinogenic, and anticoagulant activities. Moreover, it acts as a local analgesic [36].

The inhibition zones observed after 24 h were stable and identical after 48 and 72 h of incubation. EOs are less effective in the vapor disc diffusion method compared to the disc diffusion method. Thyme and clove oils had volatile components that had inhibition zones even when the disc with EO had no contact with the bacteria. Thyme had the best effect for all test microorganisms, while clove only had an effect on *S. aureus* (Table 5).

The minimal inhibitory concentration of an antimicrobial agent is the lowest (i.e., minimal) concentration of the antimicrobial agent that inhibits a given bacterial isolate from multiplying and producing visible growth in the test system. This concentration was determined by incubating a known quantity of bacteria with specified dilutions of the antimicrobial agent. Using a similar broth dilution method, Zhang et al. [53] found that MIC was 1.0 mg/mL of cinnamon for both *S. aureus* and *E. coli*. Broth microdilution is the most widely used method in clinical laboratories, but agar diffusion is also used. Due to the oily content of EOs, using solvents is needed to obtain homogeneous dilutions. The results obtained using three solvents namely Tween-20, DMSO, and ethanol by the two test methods (agar MIC testing and the broth microdilution test) are shown in Table 6. There are some differences between the test results, but they all showed that thyme is the most effective EO, with a very low concentration needed to inhibit the growth of *S. aureus* and *E. coli,* followed by clove and tea tree oils. The thyme oil MIC values with the broth dilution method was approximately 0.39%–3.13% for *S. aureus* and 1.56%–3.13% for *E. coli*. Similarly, clove MIC value range were approximately 3.13%–6.26% for *S. aureus* and 6.25% for *E. coli.* A strong antimicrobial activity of thyme was also found by Mith et al. [54] when they tested different EOs and determined MIC against both food-borne pathogens and spoilage bacteria with a broth dilution method. The MIC value ranges of tea tree oil were approximately 0.80%–25.00% for *S. aureus* and 12.5%–25.00% for *E. coli*.

TTO has gained attention in the food industry for its antimicrobial [41,42] and antifungal activity [43], and it was successfully used in respiratory or genito-urinary tract infections [44].

### 2.5. Antioxidant Activity Evaluation

**Figure 9:**
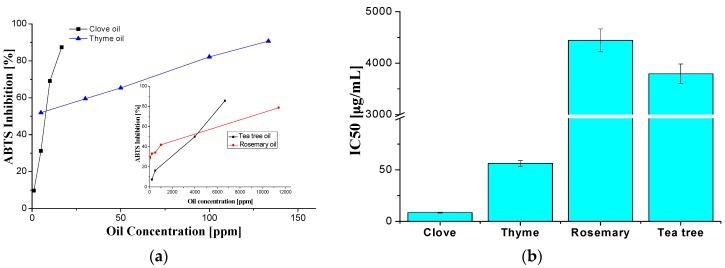
ABTS radical discoloration of studied essential oils (**a**) and the values of sample concentration required to scavenge 50% of the ABTS free radicals (IC50); (**b**) of selected essential oils.

**Figure 10:**
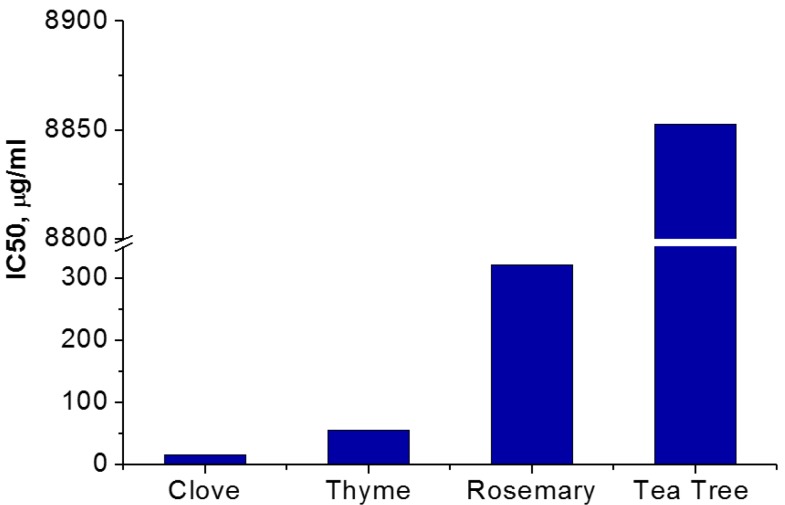
The values of the sample concentration required to scavenge 50% of the DPPH free radicals (IC50) of selected essential oils.

The results obtained by the ABTS method—Figure 9—are well correlated with those obtained by 2,2-diphenyl-1-picrylhydrazyl (DPPH)—Figure 10.

For the analyzed essential oils, IC50 is in the μg/mL range assigned to a very good antioxidant activity [55]. From the selected essential oils, the clove oil is by far the most radical scavenging active, with an IC50 of 8 μg/mL. The antioxidant activity was associated with the presence of phenolic compounds in the composition of essential oils, as determined by the Folin–Ciocâlteu method and the GC-FID technique and ORAC values. The antioxidant activity varies in the following order: clove oil > thyme oil > rosemary oil >> tea tree oil. Based on the radical scavenging tests, the clove oil is the most effective antioxidant is recommended for further such applications.

### 2.6. Preliminary Results on the Use of Encapsulated Essential Oils into Chitosan Films as Food Packaging Material

**Figure 11:**
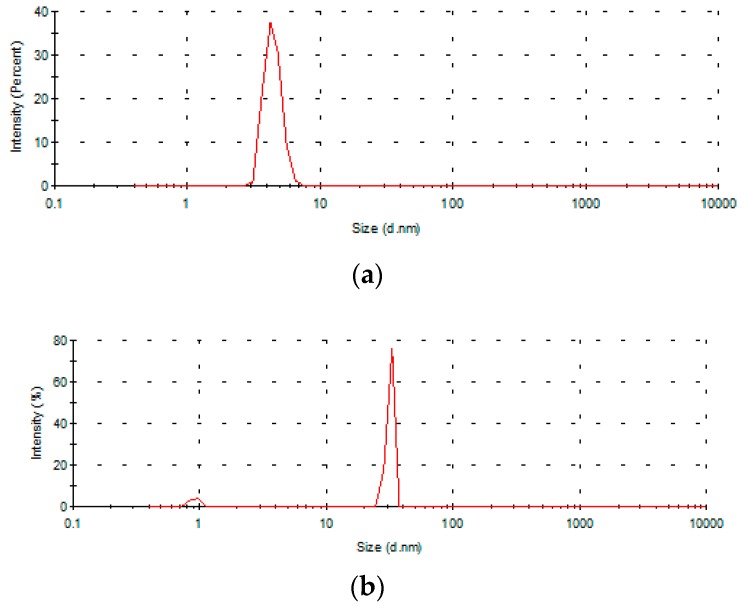
Particle size distribution of the chitosan (**a**) and chitosan/tea tree oil (**b**) emulsions in diluted acetic acid containing surfactant Tween 80.

The stability of the chitosan/essential oil emulsions was sustained by dynamic light scattering (DLS) analysis, which showed a main particle population with low dimensions (Figure 11), with the distribution showing, in the low dimension range, a single peak and a polydispersity index (PDI)-approached value of 1.0 (Table 7). The chitosan/essential oil emulsions contain two kinds of particle populations: the first one is very small, where *d* = 5 nm for the chitosan solution and 11–70 (350) nm for the emulsion containing EOs, and the second contains larger particles of 850 nm for CS and 2500–8800 nm for CS/EOs emulsions. Oils are probably concentrated around CS particles because of aggregation.

**Figure 12:**
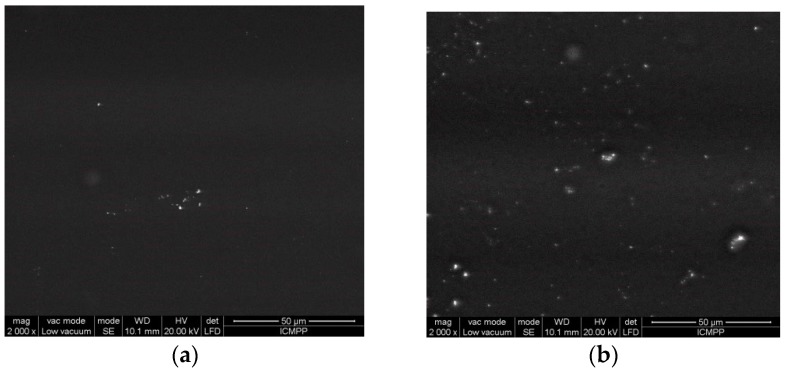
SEM images of (**a**) CS and (**b**) CS/rosemary or clove oil/T80 films at a 2000× magnification.

Scanning electron microscopy examination reveals the morphology of the films obtained by the incorporation of essential oils into chitosan films—Figure 12. The films are relatively homogeneous, and dispersion of the essential oil in the film obtained by solvent casting is homogeneous, as is evident from the SEM images taken at high magnification (2000×).

It can be observed that the surfaces are very smooth, and, by essential oil incorporation, very small particles are evidenced at high magnification. The good homogeneity of the films and the encapsulation of the oils is assured by the presence of a nonionic surfactant and emulsifier T80.

The encapsulation of active (antimicrobial and antioxidant) essential oils into the chitosan matrix leads to a significant decrease in the total number of germs for beef meat packed in such films—from 2400–1020 CFU/cm^2^ for films containing only CS to 700 CFU/cm^2^ for films containing both CS and clove oil. These films proved to have a good antimicrobial activity for delaying the spoilage of beef meat, with both antimicrobial agents acting synergistically [56]. This research will be the topic of the subsequent paper.

## 3. Experimental Section

### 3.1. Materials

#### Essential Oils

Four commercial essential oils used in this study were purchased from the Fares company (Orăştie, Romania): thyme (*Thymus Vulgaris* L.), clove (*Eugenia caryophyllus*—from dried floral buds of *Syzygium aromaticum*), rosemary (*Rosmarinus officinalis* L.), and tea tree (*Melaleuca alternifolia aetheroleum*) obtained using a distillation apparatus (according to European Pharmacopoeia 7th edition, method 8.2.12).

The Folin–Ciocâlteu phenol reagent 2N was purchased from Sigma-Aldrich (Buchs, Switzerland), and sodium carbonate decahydrate (Na_2_CO_3_·10H_2_O, *M* = 286.14) was from Chimopar S.A., Bucuresti, Romania. Methanol and gallic acid (97%, *M* = 170.12) were purchased from Sigma-Aldrich (Buchs, Switzerland).

For the determination of the antioxidant activity 2,2-diphenyl-1-picrylhydrazyl (DPPH, Sigma-Aldrich, Darmstadt, Germany), 2,2′-azino-bis(3-ethylbenzothiazoline-6-sulfonic acid) diammonium salt (ABTS, purity ≥ 98% by HPLC, Sigma-Aldrich, Darmsadt, Germany) and potassium persulfate (KPS, ACS reagent, purity ≥ 99.0%, Fluka, Buchs, Switzerland) were used. High purity solvents such as Tween-20 from Sigma-Aldrich (Buchs, Switzerland) containing lauric acid, ≥40% (balance primarily myristic, palmoyic, and stearic acids; dimethyl sulfoxide (DSMO, Sigma-Aldrich, Buchs, Switzerland), or ethanol (Sigma-Aldrich, Buchs, Switzerland) were used in the antibacterial activity test.

*Chitosan* (CS) from crab shells, with a dynamic viscosity of >400 mPa·s in 1% acetic acid (20 °C) where *MW* = 310,000–375,000 g/mol, was purchased from Sigma-Aldrich (Buchs, Switzerland).

Polysorbate 80 or Tween^®^ 80 (T80) is a viscous, water-soluble viscous yellow liquid, a nonionic surfactant and emulsifier often used in foods and cosmetics. It was offered by Sigma-Aldrich (Buchs, Switzerland) for research purposes.

### 3.2. Microorganisms

#### 3.2.1. Fungi

Three food-related fungi were used as target microorganisms in this study: *Aspergillus brasilliensis* ATCC 16404, *Penicillium corylophilum* CBMF1, and *Fusarium graminearum* G87, and they were provided from the collection of Faculty of Biotechnology of University of Agronomic Sciences and Veterinary Medicine Bucharest (Bucharest, Romania).

#### 3.2.2. Bacteria

The three tested bacterial strains were obtained from the Culture Collection at the University of Gothenburg: *Staphylococcus aureus* CCUG 1828, *Escherichia coli* CCUG 10979, and *Listeria monocytogenes* CCUG 15527. A test culture was prepared by growth on MH agar overnight, and colonies were diluted to a 0.5 McFarland in MH broth.

### 3.3. Methods of Investigation

#### 3.3.1. Determination of Total Phenolic Content in Vegetable Oils by the Folin–Ciocâlteu Reagent Method

The amount of total phenols was determined by the Folin–Ciocâlteu reagent method as described by Scalbert et al. [18]. A 10 μL volume of essential oil was dissolved in 10 mL of methanol, and 0.1 mL of methanol solution was then transferred into a volumetric flask and diluted with 0.4 mL of double distilled water and used for analysis. A Folin–Ciocâlteu reagent solution 1:10 *v*/*v* in double distilled water (1 mL) was added to the diluted methanol/oil mixture (0.5 mL), which was then mixed thoroughly and leaved for 10 minutes at room temperature. A 2 mL solution based on 15% sodium carbonate (Na_2_CO_3_·10H_2_O) was added; after 1 h of incubation at room temperature, the absorbance was measured at 740 nm with a UV-Vis 60 Cary spectrophotometer against a blank sample that was concomitantly prepared. The blank sample contained 0.1 mL of methanol without oil, 0.4 mL of water, 1 mL of a Folin–Ciocâlteu reagent solution, and 2 mL of a Na_2_CO_3_·10H_2_O solution. Gallic acid was used as the standard for the calibration curve, which was drawn using solutions of the gallic acid in methanol of different concentrations ranging between 0.01 and 1 mg/mL. Based on the measured absorbance at 740 nm, the concentration of phenolics was read (mg/mL) from the calibration line, and the total phenolics values were then expressed as mg·GAE/g·DW. Each determination was made in triplicate, and average values were reported.

#### 3.3.2. Gas Chromatography-Mass Spectrometry Detector and Flame-Ionized Detector Analysis (GC-MSD/FID)

The essential oils were characterized by gas chromatography coupled with mass spectrometry detector (GC-MSD) for qualitative analysis and, with a flame ionized detector (GC-FID) (Agilent, Santa Clara, CA, USA), quantitative analysis.

The GC analysis was performed on a 6890 Agilent Technologies chromatograph using the following parameters: a Teknokroma TR-520232 (95% dimethyl- and 5% diphenyl-polysiloxane, crosslinked; 30 m × 250 μm × 0.25 μm) capillary column, a 1 mL/min helium flow, an inlet at 175 °C, a 100:1 split ratio, and a temperature program starting at 60 °C, with a heating rate of 7 °C/min up to 200 °C and then 15 °C/min up to 300 °C, followed by isothermal heating at 300 °C for 3.33 min.

The MS detection was performed on a 5975 Inert XL Agilent Technologies detector (Agilent, Santa Clara, CA, USA), at 70 eV. Identification of chromatographic peaks was made by reference to Institute of Standards and Technology (NIST) database, with a quality of recognition above 90%.

### 3.4. Fungi Spore Suspension Preparation

To evaluate the antifungal activity of the tested essential oils, the selected fungi were grown on a Potato Dextrose Agar (PDA, Scharlau Microbiologi, Barcelona, Spain) medium in 90 mm Petri dishes for 7–9 days and stored at 25 °C. Fungal spore suspension was obtained in aseptic conditions in a laminar flow cabinet after ultraviolet sterilization for 20 min. Fungal spore suspensions were collected from the surface of fungal colonies by gentle scraping with a sterile inoculation loop and suspended in 10 mL of sterile water. Spore population was counted using a hemocytometer (Thoma camera, 0, 100 mm depth, from Hirschmann Techcolor, Eberstadt, Germany). The concentration of spore suspensions was 10^6^ spores/mL.

### 3.5. Antifungal Assay

The antifungal activity of the tested oils was determined with the disc diffusion method. Potato dextrose agar medium was poured into 90 mm Petri dishes, and, after its solidifying, in each Petri dish, 100 μL of spore suspensions (containing 10^6^ spores/mL) were spread onto the surface of the media. The dishes were left to facilitate the incorporation of fungi in the PDA medium for 30 min. Subsequently, filter paper discs (6 mm Ø; Whatman) were placed on the surface of the Petri dishes and impregnated with different amounts (volume from 2.5 to 60 µL) of essential oil. All determinations were performed in five replicates. Control plates (without essential oils) were inoculated using the same procedure. Each sample was evaluated by three analysts, and three averages of the five replicates were obtained. The final average is the arithmetic average of the three other averages. Samples were evaluated through estimation of the degree to which the Petri dish surface was covered by fungal mycelium and through an inhibition rate (IR) calculation.

The inhibition rate was calculated as follows:

(1) $$IR(\%)=\frac{C-S}{C}\times 100$$

where *C* is the rate of the fungal mycelium growth of the control, and *S* is the rate of the fungal mycelium growth of the samples.

The dishes were sealed with parafilm tape to prevent the volatilization of essential oils and incubated for 7 days at 25 °C. The efficacy of the treatment was evaluated after 7 days by the inhibition rate *IR* (%).

### 3.6. Determination of the Minimum Inhibitory Concentration (MIC) of Fungi

Essential oils were screened to determine their minimum inhibitory concentration against the food spoilage microorganisms selected. The same procedure as the one described for the antifungal assay was used to determine the minimum inhibitory concentration (MIC). The definition of the MIC differs between various publications [57,58,59], so it was decided that the MIC would be defined as the lowest volume of essential oil that inhibited the growth of the tested fungi with an inhibition rate higher than 80%. The MIC is expressed in different units including mg/mL [60], µg/mL [61,62], µL/L [63,64], µL/mL [65], and ppm [62,66]. In this study, the MIC of fungi was expressed as ppm.

### 3.7. The Agar Disc Diffusion Method

The protocol of the Clinical and Laboratory Standards Institute (CLSI) [67,68] was followed with some modifications. The bacterial suspensions were diluted to a McFarland standard of 0.5 (0.5 McFarland standard is prepared by mixing 0.05 mL of 1.175% barium chloride dihydrate (BaCl_2_·2H_2_O), with 9.95 mL of 1% sulfuric acid (H_2_SO_4_), and adjusted to about 10^4^–10^5^ bacteria/mL before use. A 0.1 mL portion from this bacterial dilution was spread on Mueller–Hinten (MH) agar (MHA, Sigma-Aldrich, Darmstadt, Germany). Subsequently, sterile paper discs (Macherey-Nagel, Düren, Germany, LOT: 141112) 6 mm in diameter were placed within 15 min onto the inoculated agar surface containing 10 µL of the essential oil or 10 µL of sterile water as control. Petri dishes were incubated at 37 °C for 24 ± 1 h. After incubation, the inhibition zones were measured as the complete inhibition in mm, as a diameter including the disc. The inhibition zones were measured in triplicate with a vernier caliper. The distance from the colonies closest to the disc to the center of the disc was doubled to obtain a diameter.

### 3.8. The Vapor Disc Diffusion Method

MH-agar plate surfaces inoculated with *E. coli* and *S. aureus* and Tryptone Soya Agar (TSA, CM 0131, Oxoid Ltd., Thermo Fisher Scientific, Basingstoke, Hampshire, UK) with *L. monocytogenes* were prepared as described in Section 3.7. A volume of 10 µL of each pure EO was added to sterile filter discs with a diameter of 6 mm, and sterile water was used as control. The discs were placed on the inside center of a Petri dish lid in a sterile working hood. A control was also prepared by adding 10 µL of sterile water to the filter discs.

The inoculated agar plate was then placed to fit the lid but with the agar upwards. This created a closed Petri dish with a distance of about 1 cm between the disc and the bacteria inoculated agar surface in accordance with Goni et al. [69]. The Petri dishes were sealed with adhesive tape to prevent volatile components of the essential oils to escape.

### 3.9. Determination of the Minimum Inhibitory Concentration (MIC) of Bacteria

#### 3.9.1. Agar MIC Testing

The preparation of agar, broth, and bacteria were carried out as described in Section 3.7. MICs was determined for thyme, clove, and tea tree against *S. aureus* and *E. coli* according to the method used by Zhang and Mith [53,54] with some minor modifications. The original solution of each EO was dissolved in Tween-20 (0.5%), dimethyl sulfoxide (DSMO, 5%), or ethanol (100%) in a 1:1 ratio to make serial 2-fold dilutions, yielding solutions with a gradient from 100% oil to 0.2% oil (*v*/*v*). A volume of 10 µL of each oil dilution was added to the 6 mm paper disc and then placed on MH agar plates with a 100 µL bacterial concentration containing a 10^4^–10^5^ CFU/mL suspension of each tested bacterium. DSMO without bacteria was used as a control.

#### 3.9.2. Broth Microdilution MIC Testing

Similar dilutions in either Tween-20 (0.5%), dimethyl sulfoxide (DSMO, 5%), or ethanol (100%) were transferred to sterile microtiter plates (honeycomb plates with 100 wells). Three parallel dilution series were used with 100 µL in each well. The microtiter plates were mounted in a Bioscreen C (Oy Growth Curves Ab Ltd., Helsinki, Finland), an automated growth curve analysis system, programmed to measure absorbance at 600 nm (abs 600 nm) at regular time intervals at 37 ± 0.1 °C. Prior to each measurement, the plates were shaken for 10 s (default setting). Growth curves of both strains were recorded. Time-to-detection (TTD) were defined as *ABS*_600nm_ = 0.2. Lack of absorbance above 0.2 were defined as growth inhibition.

### 3.10. Antioxidant Activity Evaluation

The inhibition concentration of four essential oils (thyme, clove, rosemary, and tea tree) and the stability of natural antioxidant at different concentrations were evaluated using 2,2’-azino-bis 3-ethylbenzthiazoline-6-sulfonic acid (ABTS) and 2,2-diphenyl-1-picrylhydrazyl (DPPH) radical methods.

#### 3.10.1. ABTS^•+^ (2,2’-Azino-bis 3-ethylbenzthiazoline-6-sulfonic Acid) Radical Cation Scavenging Assay

The ABTS^•+^ scavenging test is used to determine the antioxidant activity of both hydrophilic and hydrophobic compounds. ABTS^•+^ is generated by mixing 2.5 mL of 7 mM ABTS with 2.5 mL of 14.7 mM potassium persulfate (KPS) water solutions and stored in the dark at room temperature for 16 h. The reaction between ABTS^•+^ and potassium persulfate directly generates the blue green ABTS^•+^ chromophore, which can be reduced by an antioxidant, thereby resulting in a loss of absorbance at 750 nm.

The antioxidant capacity is expressed as percentage inhibition, calculated using the following formula [70]:

(2) $$Inhibition(\%)=\left[\frac{A_{\mathrm{control}}-A_{\mathrm{sample}}}{A_{\mathrm{control}}}\times 100\right]$$

where *A*_control_ is the absorbance of the ABTS radical in methanol, and *A*_sample_ is the absorbance of an ABTS radical solution mixed with the sample. All determinations were performed in triplicate.

#### 3.10.2. DPPH Radical Scavenging Assay

The DPPH radical scavenging assay for essential oils was measured using the 2,2-diphenyl-1-picrylhydrazyl (DPPH) radical. The DPPH is a stable free radical with a violet color that, under the action of proton-donating compounds, is reduced to a light yellow color, and this change can be monitored at 517 nm [71]. Concentrations of the methanolic solution between 0.156 and 20 mg/mL were tested.

The radical scavenging activity was calculated according to the following equation:

(3) $$\%RSA=100\times \left(\frac{A_{\mathrm{control}}-A_{\mathrm{sample}}}{A_{\mathrm{control}}}\right)$$

where *A*_sample_ represents the absorbance of the sample solution in the presence of DPPH, and *A*_control_ is the absorbance of the standard DPPH solution. For more details, see ref. [72].

### 3.11. The Encapsulation of Essential Oils into the Chitosan Matrix by the Emulsion/Solvent Casting Method

The film forming solutions were prepared using highly viscous chitosan from crab shells where *MW* = 310,000–375,000 g/mol, with a dynamic viscosity of >400 mPa·s in 1% acetic acid (20 °C). The chitosan film-forming solution was prepared by dissolving 2 g of chitosan per 100 mL of a 5% acetic acid aqueous solution. Food grade essential oils (clove, thyme, rosemary, and tea tree oils) were incorporated in a proportion of 0.75 mL/g chitosan, and Tween 80 (Sigma-Aldrich, Darmstadt, Germany) (0.125 g/g chitosan) was added as an emulsifying agent. The essential oil-added film-forming solution was homogenized with an ultrasonic processor UP50H (Hielscher—Ultrasound Technology, Teltow, Germany) using a power of 50 W at 30 kHz). The films were obtained by casting 50 mL in glass Petri dishes (153 cm^2^) and drying first at 25 °C in a forced-air oven for 24 h and then at 40 °C in a vacuum oven to yield a uniform thickness in all cases. Prior to analyses, the films were conditioned in desiccators for 2 days at 22 °C over a saturated solution of NaBr (58% relative humidity) [56].

### 3.12. The Dynamic Light Scattering (DLS) Analysis

The DLS analysis of the oils-in-chitosan emulsions was carried out using a Zetasizer NS (Malvern Instruments Ltd., Malvern, UK), according with standard ISO 13321 (1996). This analysis only gives a mean particle size (z-average) and an estimate of the width of the distribution (polydispersity index (PDI)). It is very sensitive to the presence of aggregates due to the inherent intensity weighting. The oil/chitosan ratio was kept at 0.75 mL/1 g chitosan. For DLS, the concentration of the initial solution was 1.5 wt % chitosan and the corresponding concentration of the oils. The chitosan/essential oil emulsions of 1.5 wt % were diluted three times prior to analysis with the 5 wt % acetic acid aqueous solution.

### 3.13. Scanning Electron Microscopy (SEM)

SEM examination was carried out using QUANTA 200 scanning electronic microscope (FEI Company, Hillsboro, OR, USA), with an integrated EDX system, GENESIS XM 2i EDAX (FEI Company, Hillsboro, OR, USA) with a SUTW detector and without any further treatments at different magnifications given on the image micrographs.

### 3.14. Statistical Analysis

Each analysis was performed in five replicates. Data were analyzed statistically with the one-way analysis of variance ANOVA test using the SPSS statistics. Significant differences between tested samples and the control were defined at *p* < 0.05.

## 4. Conclusions

Based on GC-MSD/FID analysis, it has been established that the commercially available thyme, rosemary, clove, and tea tree essential oils correspond to AFNOR/ISO standards.

The results of this study demonstrate the potential of thyme oil, clove oil, and tea tree oil for use as antifungal agents against tested fungi strains (*Fusarium graminearum* G87, *Penicillium corylophilum* CBMF1, and *Aspergillus brasiliensis* ATCC 16404) and three potential pathogenic food bacteria: *Staphylococcus aureus*, *Escherichia coli*, and *Listeria monocytogenes*. The overall results of the MIC values show that thyme oil is the most effective with a very low concentration used against both on the three fungi and bacteria, followed by clove and tea tree oils, while the rosemary oil is less active or inactive.

Concerning the antioxidant activity, the IC50 of the analyzed essential oils is in the μg/mL range that is assigned to a very good antioxidant activity which varies in the following order: clove oil > thyme oil > rosemary oil > tee tree oil, and the clove oil exhibits the highest radical scavenging activity.

Promising results were obtained by their incorporation into chitosan emulsions and films, with potential applications in food packaging that will be detailed in a future paper. Taking into account their biological activity in addition, it is expected that their use can also benefit consumers’ health.

On the basis of the obtained results, it can be concluded that these essential oils can be suitable alternatives to chemical additives, satisfying the consumer demand for naturally preserved food products and ensuring their safety at the same time.

## Figures and Tables

**Table 1:** Total phenolic content in the investigated essential oils.

Oil Type	Total Phenolic Content (mg·GAE/g·DW) *
Thyme	0.349
Clove	1.136
Rosemary	0.000
Tea Tree	0.034

* mg·GAE/g·DW (mg of gallic acid equivalent per g dry weight).

**Table 2:** The main classes of compounds found in the studied essential oils.

Class of Compounds	Main Compounds
Monoterpenes (*C10* with 2 *isoprene units*)	Borneol, Camphene, Camphor, Carene, Carvol, Citral, p-Cymene, Eucalyptol, Fenchone, Geraniol, Limonene, Linalol, Menthone, Myrcene, Ocimene, Phellandrene, Piperitol, Terpinene, Thujene, Thymol
Phenylpropanoides (*C6* with *C3 side chains*)	Apiole, Chavicol, Cinnamaldehyde, Estragole, Eugenol, Myristicin
Sesquiterpenes (*C15* with 3 *isoprene units*)	Amorphene, Aromoadendrene, Cadinene, Caryophyllene, Cubebene, Elemene, Farnesene, Globulol, Gurgujene, Isolendene, Maaliene, Panasinsen, Selinene, Spathulenol

**Table 3:** The composition of the selected commercial essential oils from the Fares Company, Romania, compared with AFNOR/ISO standards *.

Chemical Compound	Thyme	Clove	Rosemary	Tea Tree
ORAC (μTE/100 g) **	15,960	1,078,700	330	
α-Thujene	0.4			
**α-Pinene**	1.6		**11.4**	**3.9**
**Camphene**	1.0		**5.0**	
β-Pinene			9.4 (<9)	
**β-Myrcene**	**2.5**			2.5
2-Carene	2.3			10.3
**p-Cymene**	**22.5**		1.3	**2.8**
**Limonene**			**2.6**	**3.6**
**Eucalyptol**			**43.1**	**2.3**
**γ-Terpinene**	**7.9**			**16.3**
α-Terpinene				*4.1* (>5)
**Linalol**	**5.6**			
**Camphor**			**11.3**	
**Borneol**	1.1		**3.0**	
**4-Terpineol**				**38.7**
**α-Terpineol**			1.8	**4.6**
**Thymol**	**43.1**			
**Carvacrol**	**2.7**			
**Eugenol**		**85.7**		
**β-Caryophyllene**	2.3	**4.5**	3.2	2.2
α-Caryophyllene		0.9		
**Aromadendrene**				**1.1**
Ledene				1.1
**Eugenol acetate**		*7.9* (>8)		
**δ-Cadinene**				**0.9**
Caryophyllene oxide		0.4		

* The bold numbers in Table 3 indicate values within the limits of the standards; italic numbers are below the limits, while the underlined are over the limits, the bracket showing the closest limits in the standard. ** ORAC—oxygen radical absorption capacity; expressed as μmol Trolox equivalent (TE) at 100 g, accuracy of ±5%, as presented in the literature [26,27].

**Table 4:** Minimum inhibitory concentration (MIC) of essential oils tested against *Fusarium graminearum* G87, *Penicillium corylophilum* CBMF1, and *Aspergillus brasiliensis* ATCC 16404.

Essential Oil Fungal Strain	Thyme (ppm)	Clove (ppm)	Rosemary (ppm)	Tea Tree (ppm)
*Penicillium corylophilum* CBMF1	174.41	465.11	NA	930.23
*Fusarium graminearum* G87	162.79	930.23	NA	1395.34
*Aspergillus brasiliensis* ATCC 16404	116.27	697.67	NA	813.95

NA—not applicable.

**Table 5:** Mean inhibition zone diameter (mm) by the vapor disc diffusion method.

Essential Oil	Vapor Phase Method		
	*S. aureus*	*E. coli*	*L. monocytogenes*
Thyme	56.8 ± 6.9	28.3 ± 1.3	48.5 ± 4.9
Clove	14.2 ± 2.6	0 ± 0	0 ± 0
Rosemary	0 ± 0	0 ± 0	NA
Tea tree	0 ± 0	0 ± 0	0 ± 0

**Table 6:** The minimal inhibitory concentration (MIC) (%) of essential oils in three different solvents (*v*/*v*).

EO	Method	MICs Value for *S. aureus*			MICs Value for *E. coli*		
		Concentration of Oil (*v*/*v*)			Concentration of Oil (*v*/*v*)		
		0.5%	5%	100%	0.5%	5%	100%
		Tween-20	DMSO	Ethanol	Tween-20	DMSO	Ethanol
Thyme	Agar	1.56	12.50	1.56	3.13	6.25	1.56
	Broth	0.39	0.78	3.13	3.13	3.13	3.13
Clove	Agar	3.13	12.50	12.50	12.50	12.50	3.13
	Broth	6.25	3.13	3.13	6.25	6.25	6.25
Tea Tree	Agar	6.25	25.00	25.00	12.50	12.50	12.50
	Broth	0.80	25.00	25.00	12.50	25.00	25.00

**Table 7:** DLS results for essential oil/chitosan emulsions used to obtain films for food packaging.

Sample	Z First Peak, d (nm)	Z Average (nm)	PDI
Chitosan	5	850	0.997
Thyme (*Thymus Vulgaris* L.)	11.5 and 350	6190	0.735
Cloves (*Eugenia caryophyllus* from dried floral buds of *Syzygium aromaticum*)	10.5	8790	1.0
Rosemary (*Rosmarinus officinalis* L.)	60	2550	1.0
Tea tree (*Melaleuca alternifolia aetheroleum*)	15 and 70	6360	1.0
